# Preparation of Cu-ZSM-5 catalysts by chemical vapour deposition for catalytic wet peroxide oxidation of phenol in a fixed bed reactor

**DOI:** 10.1098/rsos.172364

**Published:** 2018-04-04

**Authors:** Donglin He, Huiping Zhang, Ying Yan

**Affiliations:** School of Chemistry and Chemical Engineering, South China University of Technology, Guangzhou 510640, People's Republic of China

**Keywords:** Cu-ZSM-5 catalysts, chemical vapour deposition, phenol, catalytic wet peroxide oxidation, fixed bed reactor

## Abstract

Cu-ZSM-5 catalysts were prepared by chemical vapour deposition for catalytic wet peroxide oxidation (CWPO) of phenol in a fixed bed reactor. Firstly, Cu-ZSM-5 catalysts with Cu loading of 0.5, 2, and 6 wt% were prepared and characterized by X-ray diffraction (XRD), N_2_ adsorption–desorption and X-ray photoelectron spectra (XPS). The characterization results demonstrated that CuO was uniformly dispersed on ZSM-5 with slight effect on the structure properties of the support. Then, several variables, such as the copper loading, reaction temperature, catalyst bed height and feed flow rate were investigated in the CWPO of phenol in aqueous solution at high concentration (1000 ppm). Compared with the catalyst prepared by the impregnation method, the Cu-ZSM-5 prepared by chemical vapour deposition has a better capacity of further oxidizing the intermediate organic products into carbon dioxide and water with less metal loading. Based on the Cu-ZSM-5 catalyst with Cu loading of 6 wt%, complete removal of phenol and a high TOC reduction (around 70%) have been achieved at the temperature of 80°C feed flow rate of 2 ml min^−1^ and catalyst bed height of 3 cm. Moreover, this catalyst maintained high catalytic activity after three runs with high phenol conversion (94%) under this optimum operating condition. Finally, the reaction mechanism was studied based on the intermediates detected by high-performance liquid chromatography (HPLC).

## 1. Introduction

Phenol and substituted phenols frequently appear in wastewater from coal processing, chemical pharmaceutical, textile mills, refineries and many other industries [1]. Their removal is still a significant area of research as increasingly stringent wastewater discharge regulations continue to be worldwide implemented. Due to its toxicity and poor biodegradability, phenol is usually chosen as a model compound for the treatment studies of hazardous organic pollutants. Physical separation, biodegradation, thermal destruction, and the advanced oxidation processes (AOPs) are typical methods in the treatment of phenolic effluents. Among the AOPs, the severe operation conditions of catalytic wet air oxidation (CWAO) (*P* = 20–200 bar and *T* = 200–320°C) increase the treatment cost [2], whereas catalytic wet peroxide oxidation (CWPO) demands a milder condition (20–80°C and atmospheric pressure) [3]. Unlike CWAO, in which the degradation rate is strongly limited by the mass transfer of molecular oxygen from the gas to the liquid phase, CWPO takes advantage of employing H_2_O_2_ as liquid oxidant to overcome the gas–liquid mass transfer limitations.

The catalysts used for the CWPO processes consisted of homogeneous catalysts and heterogeneous catalysts. Transition metal cations (Fe^2+^, Cu^2+^) have been applied in CWPO of phenol as homogeneous catalysts; their redox properties allow generating highly active OH radicals in the presence of H_2_O_2_ [4]. However, the homogeneous systems have limitations in catalyst separation, regeneration and secondary contamination. In contrast to the drawbacks of homogeneous catalysts, the effective and economic heterogeneous catalytic systems have recently attracted continuous interest and played a key role in the CWPO processes. Therefore, the transition metal catalysts such as Fe, Cu supported on activated carbons [5], Al_2_O_3_ [6,7], SiO_2_ [8], pillared clays [9] and zeolites [10–14] have been prepared for CWPO of phenol. The metal active component and the pore size–pore volume distributions have been found to strongly affect the reaction rate in the oxidation of phenol [15]. Copper components have been reported to have higher catalytic activity than iron in the process of CWPO [16]. Moreover, zeolites with uniform porous structures and high specific surface area can be a promising support. For the CWPO of phenol process with copper- or iron-containing catalysts, there is a critical problem that the leaching of the active metal element from the support would lessen the longevity of the catalyst. It has been investigated that pH related to this problem is affected by the concentration of intermediate products and contact time in the oxidation of phenol [10,17]. Fixed bed reactor takes advantage of reducing contact time and intermediate products concentration compared to the batch reactor [18].

Typically, impregnation, precipitation and ion exchange are three methods for the preparation of supported catalysts. The catalyst structure would be altered and sintered by the vital steps such as adsorption, drying, calcination and reduction of these ways [19], which could lead to the decrease of the active surface area of the catalyst. More recently, the chemical vapour deposition (CVD) method has been used to prepare stable and well-structured catalysts. High uniformity crystalline nanoparticles of metal and metal oxides can form onto porous supports or metallic sheets by CVD with using metal organic precursors. The ability to selectively deposit films onto a particular substrate area and any geometric shape is the another advantage of this technique. Pan *et al*. [20] used a home-made pulsed spray evaporation (PSE) CVD system to synthesize Cu_2_O thin films for catalytic oxidation of C_2_H_2_ and C_3_H_6_. This pure Cu_2_O catalyst has high efficiency for the complete oxidation of VOCs with good reusability and reproducibility. Chu *et al*. [21] prepared a copper-containing catalyst supported on porous activated carbon by metal-organic chemical vapour deposition (MOCVD) with nitrogen as the carrier gas, using copper acetylacetonate as precursor. This heterogeneous catalyst with monolayer dispersed active component showed a higher catalytic activity than the catalyst prepared by impregnation method for the catalytic wet air oxidation of phenol. Although, the catalyst prepared by CVD appears excellent activity in the oxidation of organics has been applied in the catalytic wet air oxidation of phenol. Little research deals with the CWPO of phenol with the catalyst prepared by CVD in a fixed bed. Main shortcomings of CVD are the high cost of the CVD device and waste of metal precursor [22]. A significant precursor condensation between evaporation zone and deposition zone in traditional CVD system would lead to a long deposition time and a low metal deposition [19–21,23–27]. Therefore, the evaporation zone and deposition zone were united as one reaction zone with shorter deposition time and higher efficiency in our work.

The aim of this work is to probe the feasibility of Cu-ZSM-5 catalysts prepared by CVD for CWPO of phenol in a fixed bed. Several variables, such as the copper loading, reaction temperature, catalyst bed height and feed flow rate were evaluated in the process of the phenol oxidation. Meanwhile, the catalytic efficiency of Cu-ZSM-5 catalysts developed by CVD and the catalyst prepared by the impregnation method (IM) were compared to identify the benefit of CVD method. Furthermore, the concentration of intermediates in the treated water was analysed to investigate the reaction mechanism and stability of the resulting catalysts prepared by CVD.

## 2. Experimental

### 2.1. Materials

Phenol was purchased from Guangzhou Chemical Reagent Factory. Hydrogen peroxide (H_2_O_2_, 30 wt% aqueous) was obtained from Jiangsu Qiangsheng Chemical Co., Ltd. Manganese dioxide was purchased from Shanghai Qiangshun Chemical Reagent Factory. Copper nitrate trihydrate was obtained from Guangzhou Chemical Reagent Factory. All of the chemical reagents mentioned above were analytical grade. Copper(Ⅱ) acetylacetonate or Cu(acac)_2_ (97%) was purchased from Aladdin. Commercial H-ZSM-5 zeolites (column; *d* = 2 mm, *L* = 2–3 mm; Si/Al = 80) were purchased from Nankai University Catalyst Factory. Deionized water was used in all synthesis process.

### 2.2. Preparation of Cu-ZSM-5 catalysts

Four catalysts referred to as Cu-ZSM-5 (0.5%), Cu-ZSM-5 (2%), Cu-ZSM-5 (4%) and Cu-ZSM-5 (6%) were prepared by CVD. The 0.5%, 2%, 4% and 6% mentioned above are the theoretical mass ratio of Cu in the ZSM-5 support. Commercial ZSM-5 zeolites (column; *d* = 2 mm, *L* = 2–3 mm; Si/Al = 80) were chosen as the porous support. Copper(II) acetylacetonate or Cu(acac)_2_ was used as the precursor material without further purification for the deposition of copper on ZSM-5. Targeted substrate was dried at 100°C for 12 h before the deposition experiment. 0.16–2.2 g of the fresh solid precursor and 8 g target substrate were placed and distributed evenly inside the reactor. Firstly, the mixture of precursor and support was degassed thrice under vacuum condition at room temperature with nitrogen exchange to create a high purity nitrogen atmosphere. Then the reactor treated at a evaporation temperature of 180°C for 30 min. After the solid precursor sublimes and forms Cu(acac)_2_ vapour in the reactor, the deposition temperature of 350°C was attained quickly and kept for 120 min to let precursor vapour decompose fully so that the active metal element Cu was deposited onto ZSM-5. The deposition experiment was carried out under atmospheric pressure with nitrogen atmosphere. Moreover, the pressure of the CVD reactor was kept around 0.10–0.12 Mpa. At last, in order to oxidize the deposited Cu into CuO, the samples were calcined in air with a heating rate of 1°C min^−1^ up to 550°C in a muffle furnace for 6 h. IM Cu-ZSM-5 (11%) was prepared by the incipient wetness impregnation. Commercial ZSM-5 zeolites (column; *d* = 2 mm, *L* = 2–3 mm; Si/Al = 80) are the support for Cu loading. Cu ion (copper nitrate trihydrate) concentration was adjusted to obtain 11 wt% Cu loading on the catalyst. The sample was air dried at 100°C for 12 h and calcined in air up to 550°C (6 h) using a ramp of temperature (1°C min^−1^).

**Figure 1.**
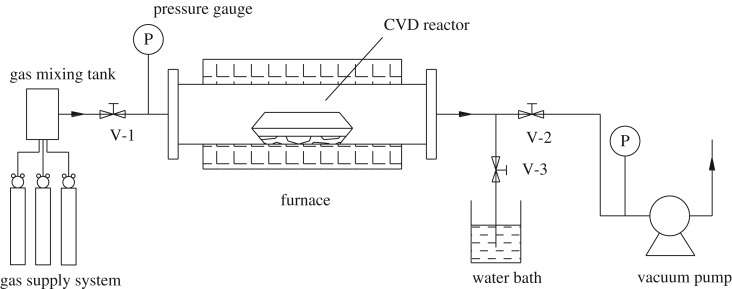
Schematic diagram of the CVD system design.

As shown in figure 1, a CVD system consisting of four major parts was used to prepare Cu-ZSM-5 catalysts. A tubular quartz tube with diameter of 100 mm and length of 1 m was placed horizontally inside the type OTF-1200X-UL single zone tube furnace. The evaporation zone and deposition zone were united as one reactor made of two porcelain boats. This system was equipped with a type GZK-101 vacuum system to achieve the vacuum condition and a type GSL-3Z-LCD gas supply system including three gas pipelines and a stainless steel gas mixing tank to control the composition and flow rate of gas. The last important component part was a water bath installed before the pump, which was used to condense the gaseous precursor.

### 2.3. Characterizations of Cu-ZSM-5 catalyst

The crystallinity and structure of Cu-ZSM-5 catalyst and copper status on ZSM-5 were studied by X-ray diffraction (XRD) techniques on a PANalytical X'Pert Pro X-ray diffractometer using Cu K*α* radiation (40 kV, 40 mA). The scanning was operated in the 2*θ* range of 5–60° with a 2*θ* step size of 1° and counting time of 10 s per step. N_2_ adsorption–desorption isotherms at 77 K were measured on an ASAP 2020 (Micromeritics Instrument Co., USA). All of the samples were out-gassed on a Micromeritics Vacrep O61 Sample Degas System at 523 K for 6 h before measurement. X-ray photo-electron spectroscopy (XPS) spectra were recorded on a Kratos Axis Ultra (DLD) instrument and using an aluminium K*a* radiation source operated at 15 kV and 10 mA.

### 2.4. Catalyst wet peroxide oxidation of phenol over Cu-ZSM-5 catalyst

**Figure 2.**
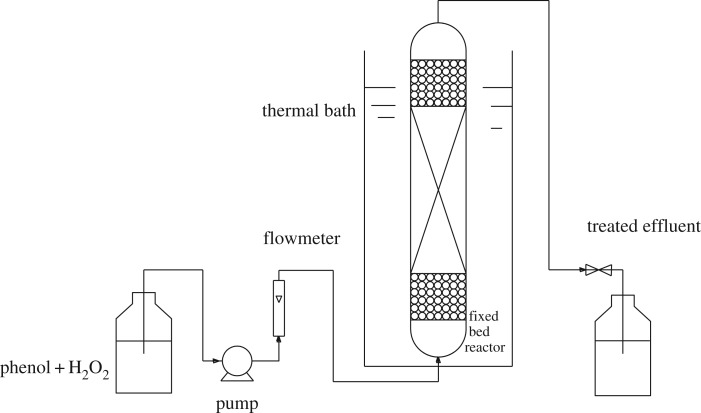
Flowchart of the experiment set-up [12].

To evaluate the catalytic activity of Cu-ZSM-5 catalysts, CWPO of phenol aqueous solution experiments were carried out in a fixed bed reactor made of a stainless steel tube (20 mm i.d., 100 mm length) under atmospheric pressure. As shown in figure 2 [12], the catalysts were fixed in the reactor between two layers of spherical inert glass particles (*d* = 2–3 mm) to improve the distribution of the inlet fluid. The aqueous solution in the feed tank including 1 g l^−1^ of phenol and 5.1 g l^−1^ of H_2_O_2_ was fed to the up-flow mode reactor by a peristaltic pump (stoichiometric amount for the total phenol oxidation according to reaction (2.1)). The temperature in the fixed bed reactor was controlled by water bath heat. More details about the fixed reactor can be seen in our previous work [12].

2.1 $${\text{C}}_{6}{\text{H}}_{5}\text{OH + 14}{\text{H}}_{2}{\text{O}}_{2}\to \text{6C}{\text{O}}_{2}+17{\text{H}}_{2}\text{O}.$$

H_2_O_2_ and TOC conversions were measured by the method mentioned in our previous paper [12]. The phenol and other organics in the treated fluid were analysed by high-performance liquid chromatography (HPLC) (Agilent 1100) equipped with a UV detector adjusted at 210 nm and an Agilent HC-C18(2) column (5 µm × 250 mm × 4.6 mm) of which the mobile phase is 1 ml min^−1^ of methanol solution (MeOH : H_2_O =  30 : 70 vol%).

## 3. Results and discussion

### 3.1. Characterization

#### 3.1.1. XRD patterns

**Figure 3.**
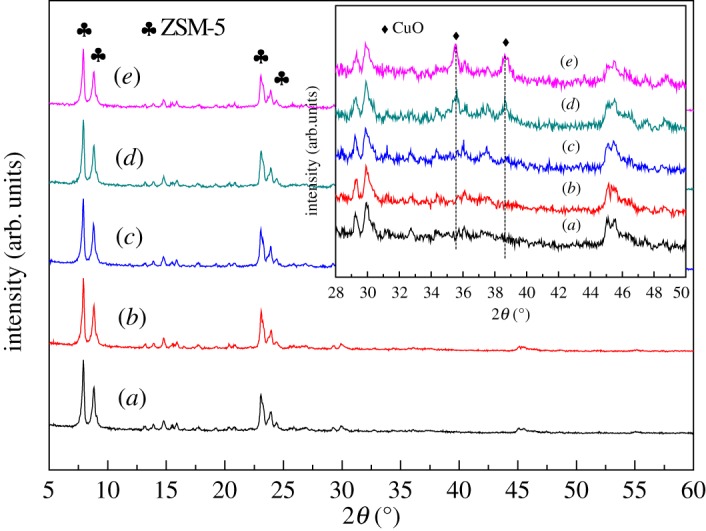
XRD patterns of the samples: (*a*) ZSM-5, (*b*) Cu-ZSM-5 (0.5%), (*c*) Cu-ZSM-5 (2%), (*d*) Cu-ZSM-5 (4%) and (*e*) Cu-ZSM-5 (6%).

As shown in figure 3, five samples gave the same diffraction peaks at the ranges of 2*θ* = 7–9° and 2*θ* =23–25°, matching well with the standard pattern of ZSM-5 according to the literature [28]. Moreover, only a slight decrease was observed in the intensity of ZSM-5 zeolite diffraction peaks. It was indicated that organized pore structure was always maintained after the CVD process.

For Cu-ZSM-5 (4%) and Cu-ZSM-5 (6%), two clear peaks of copper oxide around 35.5° and 38.7° were detected [29]. It was confirmed that CuO active component (about 24 nm) was successfully loaded on the ZSM-5 support. At Cu loading of 0.5 and 2 wt%, the peaks at 2*θ* = 35.5° and 2*θ* = 38.7° characteristic of monoclinic CuO phase were hardly detected. This result can be explained by: (i) the deposition of CuO onto the support in a monolayer distribution, or (ii) the reduction of the particle size down to the detection limit of the instrument [11,21].

#### 3.1.2. N_2_ adsorption-isotherms analysis

N_2_ adsorption–desorption isotherms of ZSM-5 and Cu-ZSM-5 catalysts are displayed in the electronic supplementary material. Meanwhile, the BET surface areas and pore properties are summarized in table 1.

**Table 1.** Properties of Cu-ZSM-5 catalysts by nitrogen adsorption. Cu content (wt%): as determined by atomic absorption spectrophotometer analysis; *S*_BET_: surface area calculated by the BET (Brunauer–Emmett–Teller) method; *V*_total_: the total pore volume estimated by analysis of the N_2_ adsorption–desorption isotherms; *V*_micro_: micropore volume calculated from *t*-plot.

sample	Cu content (wt%)	*S*_BET_ (m^2^ g^−1^)	*V*_total_ (cm^3^ g^−1^)	*V*_micro_ (cm^3^ g^−1^)
ZSM-5	0	269.1211	0.31075	0.060394
Cu-ZSM-5 (0.5%)	0.37	273.355	0.330237	0.058892
Cu-ZSM-5 (2%)	1.40	266.0273	0.301427	0.057278
Cu-ZSM-5 (4%)	3.83	263.7946	0.331950	0.054924
Cu-ZSM-5 (6%)	5.22	256.2444	0.303994	0.053773

N_2_ adsorption–desorption isotherms show that all the samples present isotherms type II with a hysteresis loop at a high relative pressure. The shape of the loop seems to be unaltered after the CuO formation, agreeing with the previously presented XRD result that the CVD process does not result in pore structure collapse. The volume adsorbed increases obviously and continually with the increasing relative pressure, which can be caused by the multilayer adsorption and indicate the existence of micropores.

As shown in table 1, the micropore volume was found to slightly decrease as the Cu loading rises, which can be explained by the filling of the micropore by the CuO phase. Therefore, some CuO particles at size lower than the micropore size of the support were formed into the micropore of the support. The surface area decreased with the increase of Cu loading while the *S*_BET_ of Cu-ZSM-5 (0.5%) was little larger than the support. It was indicated that the small CuO particles were dispersed out of the pore of the support at low Cu loading. Moreover, the increase of Cu loading would lead to the decrease of *S*_BET_ when the Cu loading was over 2 wt%. Overall, the pore volume and pore size of the supports were maintained after the CVD process, matching well with the XRD characterization.

#### 3.1.3. XPS analysis

**Figure 4.**
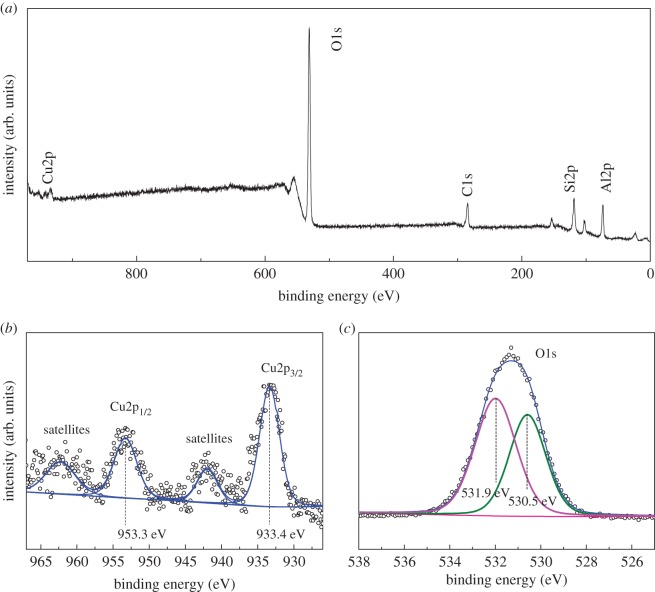
XPS analysis of the Cu-ZSM-5: (*a*) survey; (*b*) Cu 2p; (*c*) O 1s.

The surface composition and the surface copper loadings of Cu-ZSM-5 (6%) were confirmed by XPS with the binding energy positions of C 1s peak at 284.6 eV as the reference for calibration. As presented in figure 4*b*, the binding energy of 933.4 and 953.3 eV is ascribed to Cu 2p_3/2_ and Cu 2p_1/2_ of the Cu core peaks, which are closer to the positions of CuO (Cu^II^) rather than Cu^0^ [30,31]. The formation of CuO can be further confirmed by the shake-up satellite peaks around 941.9 and 961.6 eV due to the satellite effect [32]. Figure 4*c* showed that two peaks can be split from the O 1s spectrum. The O 1s peak at 530.6 eV was assigned to oxygen in CuO [33–35]. Moreover, the other around 532.4 eV may be assigned to surface adsorbed oxygen. In conclusion, the XPS results match well with XRD characterization that CuO was formed onto the support.

### 3.2. Phenol oxidation over Cu-ZSM-5 catalyst in a fixed bed reactor

#### 3.2.1. Effects of the reaction temperature

**Figure 5.**
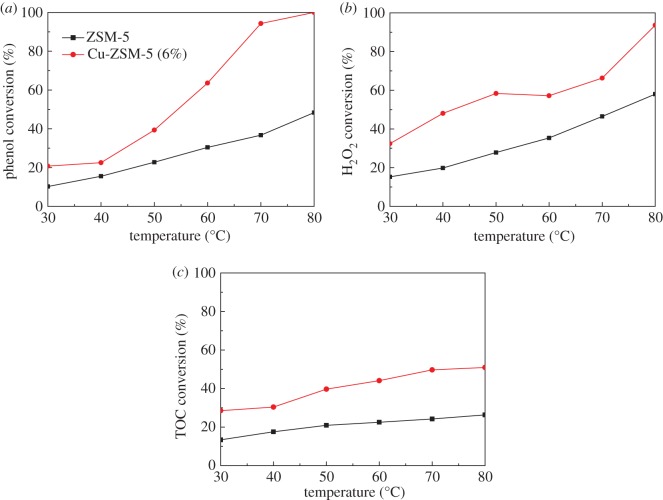
Effects of temperature on the catalytic performance: (*a*) phenol conversion, (*b*) H_2_O_2_ conversion, (*c*) TOC conversion (at feed flow rate of 2 ml min^−1^ and catalyst bed height of 2 cm, with Cu-ZSM-5 (6%)).

The behaviours of ZSM-5 and Cu-ZSM-5 (6%) for the catalytic oxidation of phenol at different temperatures (from 30°C to 80°C) were evaluated in a fixed bed reactor with the feed flow rate of 2 ml min^−1^ and the catalyst bed height of 2 cm. As shown in figure 5, the efficiencies of ZSM-5 and Cu-ZSM-5 (6%) for the CWPO of phenol were enhanced with the increase of the temperature. This was due to the fact that the H_2_O_2_ decomposition into OH· radicals was accelerated as the temperature increased [36]. For the support of ZSM-5, a large amount of hydrogen peroxide was still present at the highest temperature (80°C), reflecting the poor oxidation performance of the ZSM-5 without the active content loads. The marked increases in the conversion of phenol, H_2_O_2_ and TOC were detected as the temperature climbed with the Cu-ZSM-5 (6%). At 80°C, a total phenol conversion was achieved and a TOC conversion above 51% was reached with this catalyst. Therefore, a higher temperature and the loading of Cu were needed to improve phenol conversion and enhance oxidation rates, thus reducing the TOC of the treated effluent in CWPO experiments.

#### 3.2.2. Effects of the copper loading

**Figure 6.**
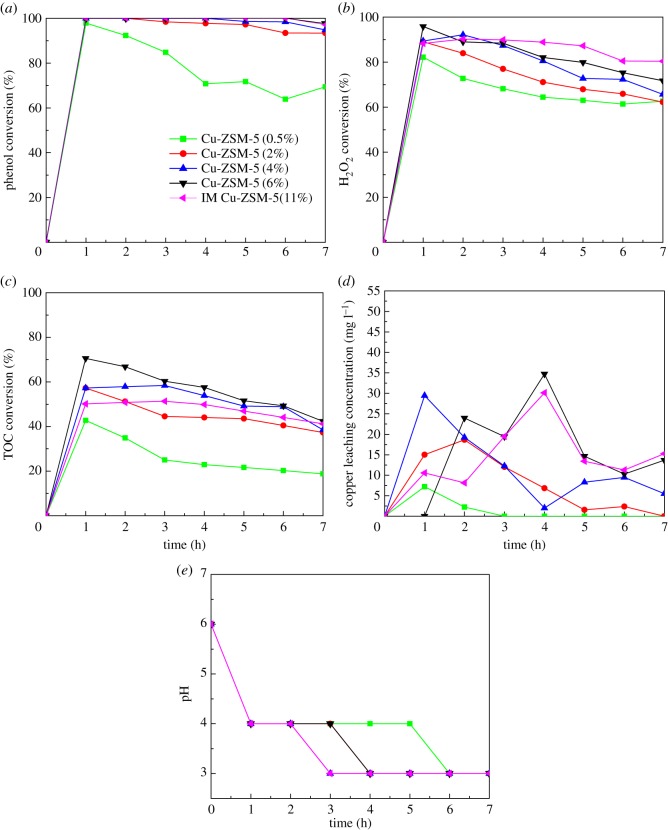
Effects of the copper loading on the catalytic performance: (*a*) phenol conversion, (*b*) H_2_O_2_ conversion, (*c*) TOC conversion, (*d*) Cu leaching concentration, (*e*) pH (at the temperature of 80°C, feed flow rate of 2 ml min^−1^ and catalyst bed height of 2 cm).

To explore the effect of the copper loading on the CWPO of phenol, several experiments were carried out with catalysts of the Cu loading ranging from 0.5 to 6 wt% in a fixed bed reactor at 80°C by using the feed flow rate of 2 ml min^−1^ and the catalyst bed height of 2 cm. The experimental results consisted of the conversion of phenol, H_2_O_2_, TOC and the Cu leaching concentration in the treated solution. As shown in figure 6*a*, the phenol conversion increased as the theoretical loading of Cu increased from 0.5 to 6 wt%. A total elimination of phenol was reached with Cu-ZSM-5 (6%) while the phenol conversion of Cu-ZSM-5 (0.5%) was only 65%. Figure 6*b* showed that the H_2_O_2_ conversion of all the catalysts diminished slightly in the 7 h perhaps due to the decrease in catalytic activity caused by Cu leaching or coke forming [37]. The same variation trend can be seen in the TOC conversion shown in figure 6*c*. It was indicated that the level of TOC conversion was enhanced with an increase in the copper loading in ZSM-5. The TOC conversion was lower than phenol and H_2_O_2_ conversion in all cases, which implied that the products of the phenol oxidation were not only carbon dioxide and water but also different intermediates. Moreover, the low pH recorded in figure 6*e* confirmed the presence of organic carboxylic acids in the treated water. Figure 6*d* indicated that the higher loss of copper ions in the liquid effluent would be detected with the larger amounts of the copper loading. However, only a slight enhancement can be seen in the efficiency of the catalyst when the Cu loading was up from 4% to 6%. These results indicate that a higher activity of the catalyst for the CWPO of phenol was attained by increasing the copper loading.

The catalytic efficiency of Cu-ZSM-5 catalysts developed by CVD was also compared with the activity of IM Cu-ZSM-5 (11%) prepared by the impregnation method. As shown in figure 6, although the phenol conversions of Cu-ZSM-5 (6%) and IM Cu-ZSM-5 (11%) are very similar, it is clear that TOC conversion is higher when Cu-ZSM-5 (6%) is used. Moreover, the catalyst prepared by the impregnation method (Cu-ZSM-5 (11%)) has a copper loading of 11 wt%, which is much higher than the Cu-ZSM-5 (6%) prepared by CVD. This may be explained in that the copper deposited onto the ZSM-5 by CVD is highly dispersed on the active surface and copper immobilized onto the ZSM-5 by impregnation stacks on each other [21]. These results demonstrate that CVD is a superior technique in developing heterogeneous catalyst supported on porous solid. Furthermore, the catalyst prepared by CVD has a better capacity of further oxidizing the intermediate organic products into carbon dioxide and water with less metal loading.

#### 3.2.3. Effects of the catalyst bed height

Once the effects of reaction temperature and the copper loading on the CWPO of phenol in the fixed bed reactor have been discussed, the next part of this work was focused on studying the influence of the residence time on the catalytic performance of Cu-ZSM-5 (6%) by modifying the catalyst bed height and feed flow rate. The influence of the catalyst bed height was studied by using the feed flow rate of 2 ml min^−1^ and packing beds with different height (2, 3 and 4 cm) at the temperature of 80°C. The conversion of phenol, H_2_O_2_ and TOC, pH, as well as the concentration of the copper leaching and intermediates produced in the phenol oxidation were monitored during the catalytic course.

**Figure 7.**
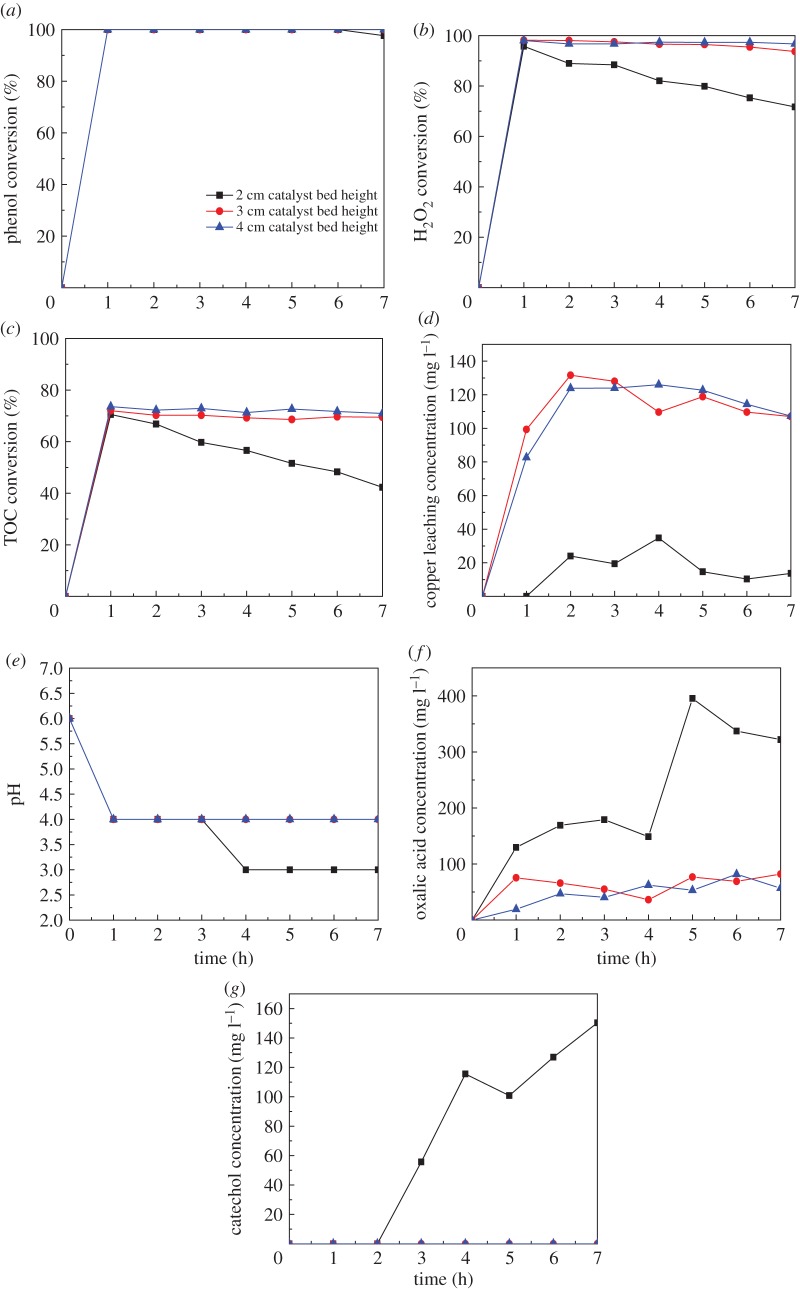
Effect of catalyst bed height on the catalytic performance: (*a*) phenol conversion, (*b*) H_2_O_2_ conversion, (*c*) TOC conversion, (*d*) Cu leaching concentration, (*e*) pH, (*f*) oxalic acid concentration, (*g*) catechol concentration (at the temperature of 80°C, feed flow rate of 2 ml min^−1^, with Cu-ZSM-5 (6%)).

As shown in figure 7*a*, phenol was almost totally removed at steady state for all the catalyst bed height (greater than 97%). However, the H_2_O_2_ conversion shown in figure 7*b* declined gradually from 95% to 72% with the catalyst bed height of 2 cm in a run. A high H_2_O_2_ conversion (97%) was achieved in a stable state when the catalyst bed height was at least 3 cm. Moreover, a similar variation trend in the TOC conversion is depicted in figure 7*c*. The enhancement of TOC conversion was evident as the catalyst bed height increased from 2 to 3 cm, being more accentuated for the longer residence time [14]. For the catalyst bed height above 3 cm, a similar or slightly higher catalytic performance was observed for the higher catalyst bed height. These results indicated the presence of organic compounds that were resistant to the degradation, even for a high catalyst bed height. Therefore, by-products remaining in the treated effluent were detected by HPLC. It is well known that many types of intermediates and final products such as benzoquinone, catechol, hydroquinone, carboxylic acids (acetic, maleic, oxalic and fumaric acids) and other oxygenated compounds (aldehydes and ketones) can be produced in the CWPO of phenol [2,38]. As for the CWPO of phenol with Cu-ZSM-5 (6%) in a fixed reactor, catechol and oxalic acid were detected in the outlet water with the catalyst bed height of 2 cm. As observed in figure 7*g*, the catechol and other aromatic compounds could be completely removed when the height of the bed increased to over 3 cm. Figure 7*f* shows that the concentration of oxalic acid that was resistant to the degradation declined as the catalyst bed height increased and diminished to about 50 ppm when the height reached 4 cm. Therefore, the presence of oxalic acids was the main reason why the low pH (about 3–4 shown in figure 7*e*) was recorded in the treated liquid effluent. It can be concluded that catalyst bed height of 3 cm turns out more suitable by taking into account the similar values of TOC removal for the packed bed of 4 cm.

#### 3.2.4. Effects of the feed flow rate

**Figure 8.**
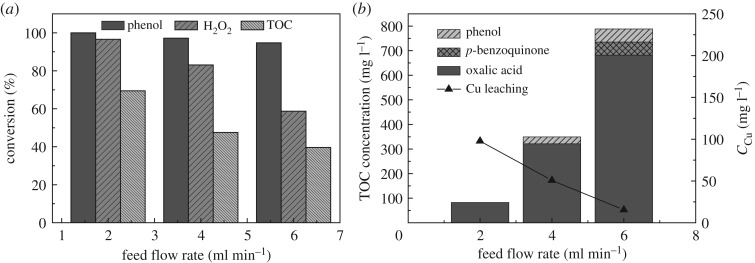
Effect of the feed flow rate on the catalytic performance: (*a*) phenol, H_2_O_2_ and TOC conversion (*b*) intermediates concentration and Cu leaching concentration (at the temperature of 80°C and catalyst bed height of 3 cm, with Cu-ZSM-5 (6%)).

The influence of the residence time was also studied by adjusting the feed flow rate of phenol solution to 2, 4 and 6 ml min^−1^ with the catalyst bed height of 3 cm at the temperature of 80°C in a fixed reactor. Figure 8*a* shows that phenol was almost completely removed for all the flow rates (above 94%). Nevertheless, the lower feed flow rate led to higher residence time, higher TOC mineralization and higher H_2_O_2_ consumption. This is because the higher residence time would encourage the generation of more hydroxyl radicals and enhance the contact between active sites of the catalyst and reactants. By-products coming from the partial oxidation of phenol were detected by HPLC analysis. As shown in figure 8*b*, high feed flow rates contributed to the increase of oxalic acid, p-benzoquinone and phenol concentration in the outlet effluent, although the existence of oxalic acid was one cause of Cu leaching. The copper leaching concentration in the treated effluent dramatically reduced with the increase of feed flow rate, giving values around 15.4–97.8 ppm. That may be because the acid resistance of CuO largely depended on the contact time instead of the acid concentration. These results indicated that the higher feed flow rate can decrease the leaching of Cu and the lower feed flow rate lets phenol oxidize more thoroughly.

#### 3.2.5. Stability and reusability of the catalysts

**Figure 9.**
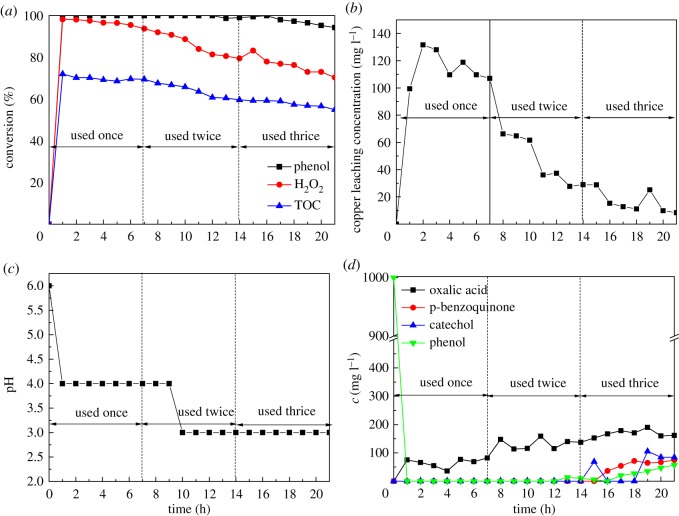
Stability tests of Cu-ZSM-5 catalyst: (*a*) conversion of phenol, H_2_O_2_ and TOC, (*b*) Cu leaching concentration, (*c*) pH, (*d*) intermediates concentration (at the temperature of 80°C, feed flow rate of 2 ml min^−1^ and catalyst bed height of 3 cm, with Cu-ZSM-5 (6%)).

To investigate the stability and reusability of the catalyst, three runs were carried out with Cu-ZSM-5 (6%) at catalyst bed height of 3 cm, temperature of 80°C and feed flow rate of 2 ml min^−1^. The used catalyst was washed three times with deionized water to remove the organics adsorbed by the catalyst and dried for 12 h at 100°C before the each run. The main parameters monitored in the course of reaction were the phenol, H_2_O_2_ and TOC conversions, the copper leaching concentration, as well as the concentration of intermediates. As shown in figure 9*a*, a high phenol removal (greater than 94%) was attained for the long-term operation. Meanwhile, a gradual decrease of the H_2_O_2_ and TOC conversion with the time was observed. The TOC conversion displaying initial values of 72% began to decrease in the twice run, which was in agreement with the decrease of H_2_O_2_ from 98% to 70%. Figure 9*b* shows that the copper leaching concentration in the treated water decreased dramatically between 7 and 11 h while the TOC conversion started a slight decrease. These results indicate that the overall catalytic performance had no direct relationship with the loss of copper species in the outlet effluent. The catalytic deactivation was found to relate with several factors: (i) the formation of carbonaceous deposits on the catalyst, (ii) poisoning of the catalyst by complexation of active sites with acid organic compounds, preventing its reactivity with other reactants, and (iii) the leaching of Cu in the liquid phase [39–41]. In order to evaluate the plausible contribution for the deactivation of the catalyst, the distribution of by-products in the treated effluent was confirmed by HPLC analysis. As shown in figure 9*d*, catechol and p-benzoquinone began to appear as the by-products after 14 h running. The oxalic acid concentration ranging from 77 ppm to 390 ppm in the treated water was in agreement with the low pH displayed in the figure 9*c*. It indicated that oxalic acid was the most refractory intermediate in the CWPO of phenol with Cu-ZSM-5 catalysts prepared by CVD and contributed to the loss of Cu content of the catalyst. Therefore, the presence of oxalic acid during the CWPO of phenol was the main factor for this deactivation. These results mentioned above suggested that Cu-ZSM-5 (6%) achieved high stability in the three runs while a slight catalyst activity decrease was observed in this oxidation process.

### 3.3. Reaction mechanism

**Figure 10.**
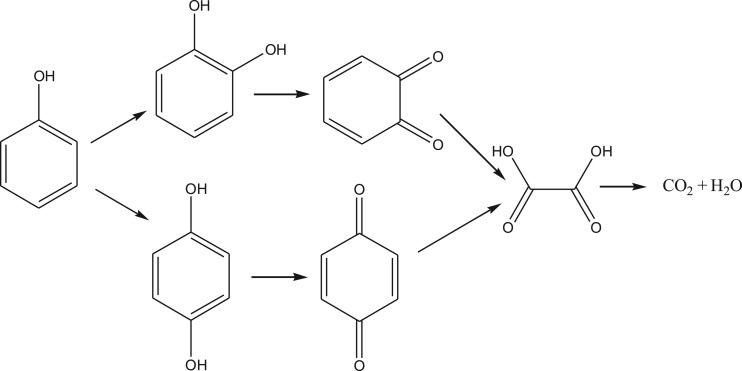
Reaction mechanism of phenol wet oxidation by hydrogen peroxide over Cu-ZSM-5 catalysts in a fixed bed.

The detection of intermediates in the CWPO of phenol is necessary to the study of the reaction mechanism of the process. The evolution of intermediates for the oxidation of phenol with Cu-ZSM-5 (6%) was monitored continually up to 21 h at the temperature of 80°C, feed flow rate of 2 ml min^−1^ and catalyst bed height of 3 cm. As shown in figure 9*d*, oxalic acid, catechol and p-benzoquinone were the identified intermediates in the outlet fluid. The aromatic intermediates were removed completely under the condition mentioned above in 14 h. As shown in figures 7*f*, 8*b* and 9*d*, oxalic acid with the concentration of 40–190 ppm was the most refractory intermediate that was difficult to remove completely. The reaction and operation condition (reaction temperature, catalyst bed height and feed flow rate) were adjusted to enhance the removal of intermediates in this work. It was found that the growth of the bed height and the decline of the feed flow rate enhanced the degradation of intermediates. The possible reaction mechanism of the CWPO of phenol in a fixed bed reactor with Cu-ZSM-5 (6%) is demonstrated in figure 10. The identified by-products imply that the oxidative breakdown of phenol is a multistep pathway. The para and ortho positions of the phenol are substituted to form catechol and hydroquinone. Hydroquinone is easily oxidized into benzoquinone with the presence of oxidants such as H_2_O_2_ and O_2_. Therefore, the present of hydroquinone in the process can be confirmed by the formation of p-benzoquinone detected by the yellow-brown colour of the treated effluent and the HPLC analysis. Oxalic acid was the main organic acids that had been identified in the process mentioned above, most of which would be partly mineralized into CO_2_ and H_2_O.

## 4. Conclusion

The Cu-ZSM-5 catalyst prepared by CVD is a promising candidate to be applied in CWPO for phenolic wastewater treatment in an up-flow fixed bed reactor. The characterization results showed that the crystal structure and pore structure properties of the support tended to remain stable with CuO uniformly dispersed on ZSM-5. The efficiency of the catalyst was improved by the increase of copper loading (from 0.5 to 6 wt%). Compared with IM Cu-ZSM-5 (11%) prepared by the impregnation method, the Cu-ZSM-5 (6%) prepared by CVD has a better capacity of further oxidizing the intermediate organic products into carbon dioxide and water with less metal loading. This demonstrates that CVD is a superior method in developing heterogeneous catalyst supported on porous solid. Based on the Cu-ZSM-5 (6%) catalyst, a complete reduction of phenol and aromatic intermediates had been achieved at the temperature of 80°C, feed flow rate of 2 ml min^−1^ and catalyst bed height of 3 cm. Moreover, a high TOC removal around 70% was achieved at steady state in a run with this catalyst in the reaction condition above. As for the subsequent stability and reusability, a high catalytic activity was maintained with a high phenol removal (greater than 94%) in three runs while it was observed to have a slight decrease. In the CWPO of phenol (1000 ppm phenol and 5.1 g l^−1^ H_2_O_2_), catechol and p-benzoquinone detected in the treated water can be removed completely with the adjustment of operation conditions. The reaction mechanism of Cu-ZSM-5 catalysts for the oxidation of phenol in a fixed bed indicated that this experimental system can reduce the production and accumulation of intermediates and attain a high-efficiency oxidation rate of phenol.

## Supplementary Material

Characterization

## Data accessibility

The datasets supporting this article have been uploaded as part of the electronic supplementary material.
