# Supplementary material for: Preparation of Cu-ZSM-5 catalysts by chemical vapour deposition for catalytic wet peroxide oxidation of phenol in a fixed bed reactor
Source: R Soc Open Sci. 2018 Apr 4;5(4):172364. doi: 10.1098/rsos.172364 (PMC5936948; doi:10.1098/rsos.172364)
Supplement: Characterization [file rsos172364supp1.docx]

**Characterization**

The suitable calcination temperature for oxidizing Cu into CuO was determined in the dry air by a Netzsch SAT-409C thermo-gravimetric (TG) analyzer from room temperature to 700 ℃ with a 10 ℃ /min temperature ramping rate. The textural and morphological information of the samples were characterized using field emission scanning electronic microscopy (FE-SEM) on a Zeiss Merlin FE-SEM. Before analysis, all of the samples were coated with an ultrathin film of platinum to make them conductive. The energy dispersive spectroscopy (EDS) were applied to analyze the chemical composition of catalysts. H_2_-Temperature programmed reduction (TPR) tests were conducted on Quantachrom Automated Chemisorption Analyzer by heating the catalyst in H_2_ (10 vol%)/ Ar flow (30 ml/ min) at a heating rate of 10 ºC / min from room temperature to 700 ºC. The hydrogen consumption was detected by thermo-conductivity detector (TCD).

Fig. S1. TGA profiles of the uncalcined Cu-ZSM-5 (6%).

The calcination temperature of Cu-ZSM-5 catalysts were determined by TG with dry air as the carrier gas. The DSC curve shown in Figure. S1 indicated that the oxidation of the supported Cu started at 330 ℃. It can be concluded from the weight-loss curve that 550 ℃ was a suitable calcination temperature for the formation of high purity CuO.

Fig. S2. N_2_ adsorption–desorption isotherms of the samples at 77K: (a) ZSM-5, (b) Cu-ZSM-5 (0.5%), (c) Cu-ZSM-5 (2%), (d) Cu-ZSM-5 (4%) and (e) Cu-ZSM-5 (6%).


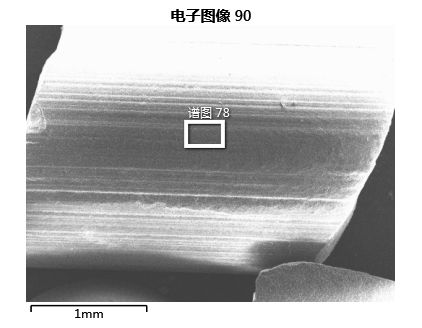


(a)


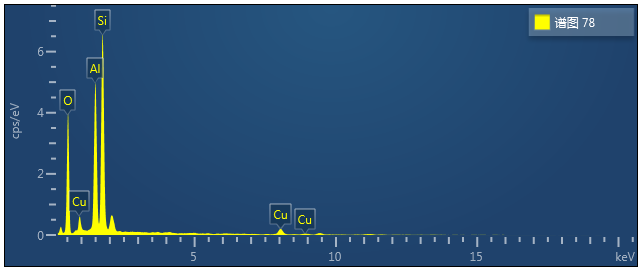


(b)

Fig. S3. Elemental analysis results of Cu-ZSM-5 (6%)

Table. S1. Elemental analysis data of Cu-ZSM-5 (6%).

| Element | wt% |
| --- | --- |
| O | 42.68 |
| Al | 19.39 |
| Si | 31.33 |
| Cu | 6.59 |
| Total | 100.00 |

Fig. S4. H_2_ temperature-programmed reduction profiles observed for (a) H-ZSM-5 support, (b) Cu-ZSM-5 (0.5%), (c) Cu-ZSM-5 (2%), (d) Cu-ZSM-5 (4%), (e) Cu-ZSM-5 (6%).

TPR experiments were carried out on Cu-ZSM-5 with 0.5, 2, 4 and 6 wt. % of Cu loading. No obvious reduction peaks are observed in TPR profiles of ZSM-5 support, indicating the support is non-reducible. The result of TPR analysis shows that Cu oxides supported on the support were reduced by hydrogen both in one step directly to metallic Cu^0^ according to reaction (S1). As reported in the Ref. [1] that pure CuO has the reduction peak at 330 ºC. All the copper loaded catalysts prepared by CVD have a much lower reduction temperature than pure CuO. This confirmed that the strong interaction among the support and copper oxide leads to easier reduction of CuO. With the Cu loading increasing, the intensities of the reduction peaks increase, and the T_max_ of the peaks have a slight decline. Generally, small particles are expected to be reduced at lower temperature. These results indicates that the Cu-ZSM-5 (6%) may have higher efficiency for the CWPO of phenol than the catalysts with lower Cu loading.

CuO + H_2_ → Cu + H_2_O (S1)

References:

[1] S. Zeng, W. Zhang, S. Guo, H. Su. Inverse rod-like CeO_2_ supported on CuO prepared by hydrothermal method for preferential oxidation of carbon monoxide. Catalysis Communications 2012;23:62-66. doi: 10.1016/j.catcom.2012.02.028
